# Cleavage-Mediated Regulation of Myd88 Signaling by Inflammasome-Activated Caspase-1

**DOI:** 10.3389/fimmu.2021.790258

**Published:** 2022-01-05

**Authors:** Monika Avbelj, Iva Hafner-Bratkovič, Duško Lainšček, Mateja Manček-Keber, Tina Tinkara Peternelj, Gabriela Panter, Steven P. Treon, Boris Gole, Uroš Potočnik, Roman Jerala

**Affiliations:** ^1^ Department of Synthetic Biology and Immunology, National Institute of Chemistry, Ljubljana, Slovenia; ^2^ EN-FIST Centre of Excellence, Ljubljana, Slovenia; ^3^ Dana-Farber Cancer Institute, Harvard Medical School, Boston, MA, United States; ^4^ Centre for Human Molecular Genetics and Pharmacogenomics, Faculty of Medicine, University of Maribor, Maribor, Slovenia; ^5^ Laboratory of Biochemistry, Molecular Biology and Genomics, Faculty of Chemistry and Chemical Engineering, University of Maribor, Maribor, Slovenia

**Keywords:** Myd88, caspase-1, inflammasomes, regulation, innate immunity, inflammation

## Abstract

Coordination among multiple signaling pathways ensures an appropriate immune response, where a signaling pathway may impair or augment another signaling pathway. Here, we report a negative feedback regulation of signaling through the key innate immune mediator MyD88 by inflammasome-activated caspase-1. NLRP3 inflammasome activation impaired agonist- or infection-induced TLR signaling and cytokine production through the proteolytic cleavage of MyD88 by caspase-1. Site-specific mutagenesis was used to identify caspase-1 cleavage site within MyD88 intermediary segment. Different cleavage site location within MyD88 defined the functional consequences of MyD88 cleavage between mouse and human cells. LPS/monosodium urate–induced mouse inflammation model corroborated the physiological role of this mechanism of regulation, that could be reversed by chemical inhibition of NLRP3. While Toll/interleukin-1 receptor (TIR) domain released by MyD88 cleavage additionally contributed to the inhibition of signaling, Waldenström’s macroglobulinemia associated MyD88^L265P^ mutation is able to evade the caspase-1-mediated inhibition of MyD88 signaling through the ability of its TIR^L265P^ domain to recruit full length MyD88 and facilitate signaling. The characterization of this mechanism reveals an additional layer of innate immunity regulation.

## Introduction

The innate immune response is essential for the defense against pathogens and supports the repair of tissue damage *via* sensing danger signals. Precise regulation of the innate immune response is of great importance in maintaining a balance between adequate defense against pathogens and damage caused due to excessive inflammation. Pattern-recognition receptors (PRRs) signal through distinctive signaling pathways, but they also affect and regulate each other to generate an appropriate response (1). Toll-like receptors (TLRs) recognize pathogen-derived ligands that induce signal transduction mediated by signaling adapters MyD88 and TRIF. Activation of the MyD88-dependent pathway results in the production of proinflammatory cytokines (2–5). MyD88 is the central adaptor of TLR signaling in all TLRs except TLR3 (3). MyD88 couples TIR-domain mediated receptor activation *via* its TIR domain to signaling kinases that are recruited through death domains (DD) of MyD88 into a myddosomal complex (6). Alternatively spliced MyD88S form, lacking the INT domain can inhibit MyD88 mediated signaling (7). On the other hand, a point mutation within TIR domain of MyD88 can lead to the constitutive activity of MyD88 that is a survival signal for proliferation in DLBC lymphoma cells, particularly in Waldenstrom’s macroglobulinemia (8–10). Inhibition of mutated MyD88 mediated signaling, on the other hand, can prevent replication of cancer cells, therefore inhibition of MyD88 signaling may be important for cancer, autoimmune disease and response to infection. Although the role of MyD88 has been first identified more than 20 years ago (11), some mechanisms of its negative regulation remain unknown.

Production of one of the most potent proinflammatory cytokines interleukin-1β (IL-1β) is highly regulated, since it requires two signals for activation: the first signal typically results from activation of TLRs and triggers production of pro-IL-1β, while the second signal leads to the activation of an inflammasome-dependent caspase-1 that is required for the processing of pro-IL-1β into a biologically active mature form (12). The pathology of several autoinflammatory diseases is linked to excessive IL-1β production (13–15). IL-1β activates the IL-1 receptor (IL-1R), which also signals through the MyD88 pathway, resulting in a positive feedback loop and amplified production of inflammatory mediators (12, 16). Therefore, tight regulation of TLR, IL-1R, and NLR signaling must ensure proper level of IL-1β.

Innate immune response is regulated on several levels, from transcriptional and epigenetic regulation (17), protein inhibitors [e.g., SARM (18) and MyD88s (19)] to proteolytic cleavage of signaling components (20). Since MyD88 is a central adaptor in TLR and IL-1R signaling, we aimed to investigate whether there is an additional mechanism of regulation involving MyD88 that could restrict the excessive signaling. The feedback regulation of signaling adaptors by inflammasome-associated caspases could have a profound physiological relevance on cell signaling in the immune response.

We hypothesized that the inflammasome activation could lead to an upstream inhibition of TLR signaling in case of MyD88 cleavage by an inflammasome-activated caspase. Bioinformatics analysis using the CASVM server (21) revealed that the intermediary domain of MyD88 comprises a potential caspase cleavage site, prompting experimental validation. Stimulation of PMA-primed THP-1 macrophages with different TLR and NLRP3 inflammasome agonists resulted in a decreased secretion of IL-6 and tumor necrosis factor-α (TNFα). Furthermore, the cleavage fragment of MyD88 was indeed observed in cells stimulated simultaneously by TLR/NLRP3 inflammasome agonists. Both human MyD88 and mouse MyD88 were cleaved by caspase-1 and, interestingly, the C-terminal TIR domain-containing cleavage fragment inhibited TLR4/MD-2 mediated signaling. The identification of caspase-1 cleavage site was confirmed by the resistance of MyD88 cleavage site mutation to proteolysis, which also prevented a decrease in MyD88-mediated cytokine production. MyD88 cleavage fragment was also observed in lymphoplasmacytic cells associated with the constitutively active MyD88^L265P^ mutation. In addition, a mouse model of inflammasome activation with lipopolysaccharide (LPS)/monosodium urate (MSU) demonstrated a decreased level of IL-6 in blood serum in contrast to LPS stimulation alone, thereby supporting the potential physiological role of the MyD88 cleavage-mediated negative feedback regulation.

## Materials and Methods

### Materials

Plasmids used in luciferase and immunodetection assays: Plasmids coding for firefly luciferase under the nuclear factor-κB (NF-κB) promoter (pELAM-1 luciferase), hMD-2 (pEFBOShMD-2), and hMyD88 (pcDNA3 hMyD88-AU1) were a kind gift from C. Kirschning, Institute for Medical Microbiology, University of Duisburg-Essen, Essen, Germany. Plasmid coding for firefly luciferase under the interferon-β promoter (IFNβ-luciferase) was a kind gift from J. Hiscott, Departments of Microbiology and Medicine, McGill University, Montreal, QC, Canada. Plasmid coding for hTLR4 (pcDNA3-hTLR4) was a kind gift from T. Espevik, Institute of Cancer Research and Molecular Medicine, Norwegian University of Science and Technology, Trondheim, Norway. Plasmid with constitutive *Renilla* luciferase (phRL-TK) was obtained from Promega, pcDNA3 from Invitrogen and plasmid pUNO1-hCASP1a from Invivogen. Plasmid pcDNA3.1-mMyD88 was a gift from Ruslan Medzhitov (Addgene #13092). Point or deletion mutants of human or mouse MyD88 were prepared either with side-directed mutagenesis or Gibson assembly (22), and primer sequences are available upon request.

TLR and inflammasome agonists used in luciferase or cytokine detection assays: *Staphylococcus aureus* subsp*. aureus* Rosenbach was obtained from ATCC (25923™), LPS *Escherichia coli* from Sigma, Pam3CSK4 and PMA from Invivogen, Alum from Thermo, ATP and nigericin from Sigma, silica from Invivogen, cholera toxin subunit B from Sigma, ultra-pure LPS *E. coli* O111:B4 and pancaspase inhibitor z-VAD-FMK from Invivogen, NLRP3 inhibitor MCC950 from Avistron, and recombinant human IL-1β from RD Systems.

### Cell Cultures

Human HEK293, HEK293T, and THP-1 cell lines were obtained from the European Collection of Cell Cultures (ECACC). MyD88-deficient cells (HEK293-I3A) were a kind gift from George R. Stark, Department of Molecular Genetics, Lerner Research Institute, Cleveland, OH. MyD88 knockout HEK293 (MyD88KOHEK293) cells were made using CRISPR/Cas9 technology. Briefly, a gRNA target sequence was selected using the CRISPR Design Tool (Zhang Lab, MIT 2015) in the first exon of MyD88 (GTTCTTGAACGTGCGGACACAGG) and cloned into pX330-U6-Chimeric_BB-CBh-hSpCas9 [a gift from Feng Zhang, Addgene 42230 (23)]. HEK293 cells were then transfected with generated plasmid and single clone colonies tested for deletion of MyD88.

Peripheral blood mononuclear cells (PBMCs) were isolated from healthy donors according to the SepMate™ protocol (Stemcell Technologies, Vancouver, BC, Canada) and stored in 10% dimethyl sulfoxide (DMSO) and FBS at –80°C. Human blood was handled according to the health guidelines, and the collecting and handling of human blood was approved by the Republic of Slovenia National Medical Ethics Committee (0120-61/2021/3). For the experiments, the cells were thawed and incubated in Roswell Park Memorial Institute-1640 (RPMI-1640) culture medium supplemented with 10% fetal bovine serum (FBS).

Peritoneal mouse macrophages were isolated from C57BL/6 mice pretreated 4 days with 1 ml of 3% thioglycolate broth (Sigma). Mouse bone marrow–derived macrophages (BMDMs) were generated by isolation of bone marrow of C57BL/6 mice and differentiation using M-CSF (e-Bioscience; 40 ng/ml) for 6 days in RPMI-1640 supplemented with 20% FBS. HEK293, HEK293T, and MyD88KOHEK293 cells were cultured in minimal Dulbecco’s modified Eagle medium (DMEM; Invitrogen), while THP-1, PBMCs, MWCL-1, and mouse peritoneal macrophages were cultured in RPMI-1640 supplemented with 10% heat-inactivated FBS (Gibco). THP-1 differentiation with PMA (10 ng/ml) for 48 h was performed using the optimized protocol from the literature (24).

CRISPR/Cas9 generated GSDMD-ko immortalized bone-marrow derived macrophages and corresponding WT control were a kind gift of Jonathan C. Kagan, Boston Children’s Hospital and Harvard Medical School, Boston, USA (25). Cells were maintained in DMEM supplemented with 10% FBS.

### Luciferase Assay

For dual luciferase assays, HEK293, HEK293-I3A or MyD88KOHEK293 cells (2*10^4^ cells/well) were plated onto 96-well white plates with clear bottom (Corning Incorporated). The cells were transfected with either expression plasmids for human, mouse MyD88 or point/deletion mutants; hCASP1a as well as firefly luciferase under NF-κB promotor (pELAM-1 luciferase) and *Renilla* luciferase (phRL-TK luciferase) for normalization using PEI transfection reagent. Six hours after transfection, cells were optionally stimulated for 16 h with the appropriate agonist, lysed in passive lysis buffer (Promega), and measured for luciferase assay.

### Immunodetection of MyD88 Cleavage in an Overexpression System

HEK293T cells (2.5*10^5^ cells/well) were plated onto 6-well plates (TPP) and transiently transfected with indicated plasmids (human, mouse MyD88 or point mutants w/o caspase-1 using PEI transfection reagent for 48 h. Cells were then washed in ice cold phosphate-buffered saline (PBS) and lysed in RIPA buffer with phosphatase inhibitors (Calbiochem). Thirty micrograms of total cell protein were loaded onto sodium dodecyl sulfate polyacrylamide gel electrophoresis (SDS-PAGE) and transferred onto nitrocellulose membrane. Nonspecific sites were blocked using iBlock (Thermo Fisher). For MyD88 detection, primary anti-MYD88 (ab2) antibody produced in rabbit (Sigma) and secondary goat polyclonal to rabbit immunoglobulin G (IgG) horseradish peroxidase (HRP; Abcam) antibodies were used. Cleaved caspase-1 was detected in supernatants after overnight methanol precipitation at –80°C using anti-caspase-1 (p20 Bally-1) antibody (Adipogen) and goat anti-mouse IgG-HRP antibody (SantaCruz).

### Cytokine Quantification/Immunodetection

THP-1 cells (1*10^5^/well) were seeded into 96-well plate and differentiated with 5 ng/ml PMA for 2 days. Cells were washed twice with RPMI and incubated in RPMI supplemented with FBS for an additional day. PMA-primed THP-1 cells were first stimulated for 4 h with the indicated TLR agonist and then for an additional 2 h with the indicated inflammasome agonist. Inflammasome inhibitors were optionally added 2 h before inflammasome stimulation. In *S. aureus* stimulation assay, overnight culture of *S. aureus* was washed using PBS and the calculated amount of *S. aureus* for indicated MOI added to cells for 4-6 h. Inhibitors were added 2 h prior to *S. aureus*. The supernatants were collected and the levels of cytokines hIL-1β, hTNF-α and hIL-6 were determined by enzyme-linked immunosorbent assay (ELISA) using Ready-Set-Go ELISA kits from eBioScience (San Diego, CA, USA). Experiments on PBMCs were performed similarly. After thawing, 1*10^5^/well viable cells were seeded into 96-well plate and left overnight in RPMI supplemented with 10% FBS. The next day, cells were stimulated similarly as THP-1 cells. Cytokine quantification on MWCL-1 was carried out after 24 h of incubation of washed cells with the indicated inhibitors. No stimulation was needed, since cells secrete cytokines constitutively (26).

Immunodetection of human MyD88 was carried out using primary anti-MYD88 (ab2) antibody produced in rabbit (Sigma) or MyD88 (D80F5) rabbit mAb (Cell Signaling Technology), phosphorylation of ERK kinases with phospho-p44/42 MAPK (Thr202/Tyr204) E10 mouse monoclonal Ab (Cell Signaling Technology), caspase-1 by anti-caspase-1 (p20 Bally-1) antibody (Adipogen), and tubulin by α/β-tubulin rabbit polyclonal Ab (Cell Signaling Technology) or actin by β-actin (8H10D10) mouse mAb (Cell Signaling Technology) with appropriate secondary antibodies, namely secondary goat polyclonal to rabbit IgG (HRP; Abcam) or goat anti-mouse IgG-HRP antibody (Santa Cruz).

BMDMs (1*10^5^ per well) were seeded into a 96-well plate. The BMDMs were primed for 3 h with ultra-pure LPS (10 ng/ml); following this, activators (1 mM ATP, 2 μM nigericin, 0.5 mg/ml Alum) were added and cells were stimulated for an additional 3 h. Cytokines were detected in supernatants with Ready-Set-Go ELISA kits from e-Bioscience/Invitrogen.

For detection of mouse MyD88 on Western blot, isolated mouse peritoneal macrophages (9 × 10^5^) were seeded into the wells of a 24-well plate. Cells were first primed with ultra-pure LPS (200 ng/ml) for 6 h; following this, activators were added (0.1 mg/ml SiO_2_, 0.5 mg/ml Alum; 20 μg/ml CTB + 2 μg/ml ultrapure LPS). After the defined time, cells were lysed, and MyD88 was detected on Western blot using primary anti-MYD88 (ab2) antibody produced in rabbit (Sigma).

### Lactate Dehydrogenase Activity Assay

The experiments were performed as in the “*Cytokine Quantification/Immunodetection*” section. Supernatants were analyzed for the presence of lactate dehydrogenase (LDH) activity. Samples were mixed with lactate in Tris buffer, pH 8.2, and a mixture of phenazine methosulphate, NAD, and iodonitrotetrazolium chloride. Supernatant from 0.1% Triton X-100 treated cells was used as positive control. Absorbance at 490 nm was measured using a SinergyMx (Biotek) multiplate reader. LDH release in percent was calculated using the supernatant of untreated cells as negative control and Triton X-100 treated supernatant as 100% LDH release.

### Animal Experiments (Cytokine Quantification in Plasma Samples and Western Blot Analysis of mMyD88 in Peritoneal Lavage)

C57BL/6 OlaHsd mice were purchased from Harlan (Italy), while *Trif* ^-/-^ knockout mice (B6.129-TRIF^tm1Aki^) were backcrossed to a single knockout from *MyD88/Trif* DKO obtained as a kind gift from S. Akira [Department of Host Defense, Research Institute for Microbial Diseases (RIMD), Osaka University, Japan]. Eight- to 12-week-old male and female mice were used for the experiments. All animal experiments, as well as isolations of peritoneal macrophages and BMDMs, were performed according to the directives of the EU 2010/63 and were approved by the Administration of the Republic of Slovenia for Food Safety, Veterinary Sector and Plant Protection of the Ministry of Agriculture, Forestry and Foods, Republic of Slovenia (Permit Number U34401-36/2015/2, U34401-3/2018/4, U34401-4/2017/4). Laboratory animals were housed in IVC cages (Techniplast), fed standard chow (Mucedola) and tap water was provided ad libitum. Mice were maintained in 12-12 hour dark-light cycle. All animals, used in the study, were healthy; accompanied with health certificate from the animal vendor.

Inflammasome activation in mice was triggered by the intraperitoneal injection of monosodium urate crystals at 1 mg/animal. For NLRP3 inflammasome inhibition, animals were given MCC950–sodium salt (50 mg/kg of body weight; Avistron) *via* intraperitoneal administration 1 h prior to MSU injection. Two hours after MSU injection, animals were administered LPS from *E. coli* O55:B5 (Sigma). Each mouse received 0.75 mg/kg of body weight of LPS intraperitoneally. After 4 h, animals were humanely sacrificed and peritoneal lavage fluid and blood were collected. Serum was prepared by centrifuging the blood for 30 min at 4°C at 3,000 rpm (Sarstedt), and cytokines were detected using Ready-Set-Go ELISA kits from eBioScience/Invitrogen. Peritoneal lavages were centrifuged and cells lysed in RIPA buffer. For detection of mMyD88 primary anti-MYD88 (ab2) antibody produced in rabbit (Sigma) and secondary goat polyclonal to rabbit IgG (HRP; Abcam) were used.

### Statistical Analysis

Statistical analysis was carried out using a one-tailed, unpaired *t* test if not otherwise noted. In case of *in vivo* studies unpaired two-tailed t-test was used and unpaired two-tailed t-test with Welch’s correction for comparison of populations of different variance was used to compare the groups treated with LPS and MSU/LPS in TRIF-deficient mice.

## Results

### NLRP3 Inflammasome Activation Downregulates LPS Signaling

**Figure 1:**
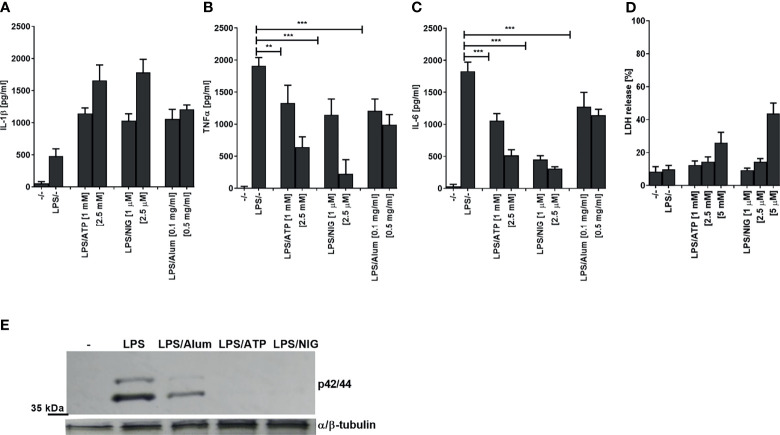
Activation of NLRP3 inflammasome decreases LPS-induced TNFα and IL-6 and ERK signaling. PMA-primed THP-1 macrophages were stimulated with LPS (10 ng/ml) for 4 h and NLRP3 agonists for 2 h. After treatment, supernatants were analyzed for cytokine secretion using ELISA **(A–C)** and LDH activity **(D)**, whereas cell lysates were used for ERK phosphorylation detection **(E)**. Data are represented as mean ± SD of at least 3 replicates **(A–D)**. Experiments were repeated at least three times with similar results. One-tailed unpaired t-test was used for statistical analysis, **p < 0.01, ***p < 0.005.

When testing inflammasome activation in human PMA-differentiated THP-1 cells by measuring IL-1β release (**Figure 1A**), we observed a decreased production of TNFα and IL-6 after stimulation of cells with a TLR4 ligand LPS in a combination with low concentrations of several NLRP3 agonists (ATP, nigericin, Alum) compared to LPS stimulation alone (**Figures 1B, C**). The observed effect on LPS signaling was not due to cell death as LDH release (a marker of necrotic cell death such as pyroptosis) was not increased and hence cell viability has not been significantly impaired under the experimental conditions (**Figure 1D**). Of note, multiple studies showed previously that NLRP3 inflammasome activation does not always lead to cell death (25, 27–29). Therefore, the activation of NLRP3 inflammasome but not pyroptosis was responsible for the decreased production of TNFα and IL-6. In addition to the impaired cytokine level, a decrease in ERK kinase phosphorylation was observed in the presence of NLRP3 agonists (**Figure 1E**). Similar results were also observed in human PBMCs (**Supplementary Figures 1A–C**).

**Figure 2:**
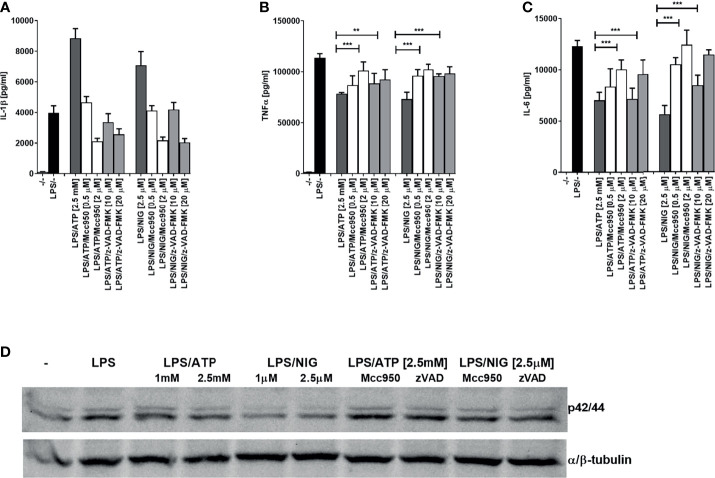
Decrease in TLR4 signaling after TLR/NLRP3 activation is reversed by caspase or NLRP3 inhibitors in PMA-primed THP-1 cells. PMA-primed THP-1 macrophages were stimulated with LPS (10 ng/ml) for 4 h and NLRP3 agonists for 2 h. Inhibitors were added 2 h before NLRP3 agonists. Supernatants were collected and levels of cytokines determined using ELISA. Data are represented as mean ± SD of at least 3 replicates **(A–C)**. PMA-primed THP-1 macrophages were stimulated as in **(A–C)** followed by another stimulation with LPS (10 ng/ml) for 30 min. Cell lysates were used for ERK phosphorylation detection **(D)**. Experiments were repeated at least three times with similar results. One-tailed unpaired t-test was used for statistical analysis, **p < 0.01, ***p < 0.005.

NLRP3 inflammasome assembly triggers proteolytic self-activation of caspase-1 which can be inhibited with NLRP3-specific inhibitor MCC950 (30–32). We were interested whether the observed decrease in cytokine production depends on inflammasome activation. While IL-1β production was inhibited by NLRP3 inhibitor MCC950 and by a pancaspase inhibitor, the production of TNFα and IL-6 on the contrast increased by the addition of inhibitors, reversing the effect of inflammasome activation (**Figures 2A–C**). In addition, similar effect of the increased activation by NLRP3 and caspase inhibition was found for the ERK kinase phosphorylation (**Figure 2D**).

### Inflammasome Activation Dampens TLR-Mediated MyD88 Signaling in Human and Mouse Cells

**Figure 3:**
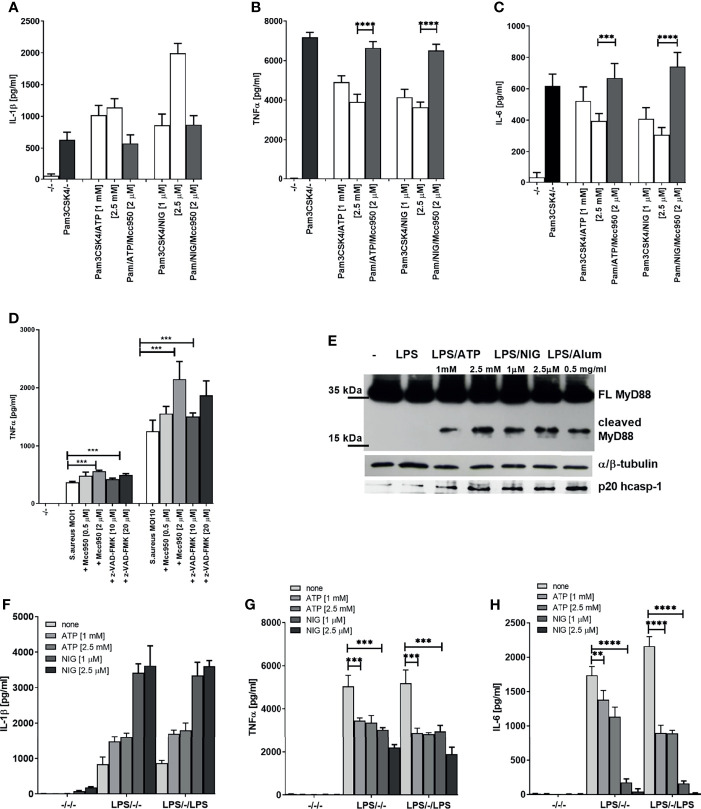
Decrease in TLR2 signaling after TLR/NLRP3 activation or *S.aureus* infection is reversed by caspase/NLRP3 inhibition. PMA-primed THP-1 macrophages were stimulated with Pam3CSK4 (0.5 µg/ml) for 4 h and NLPR3 agonists for 2 h. Inhibitors were added 2 h before the inflammasome agonist **(A–C)**. *S. aureus* was added 2 h after inhibitors, at the same time as NLRP3 activators and supernatants were sampled 6 h later **(D)**. Supernatants were collected and levels of cytokines determined using ELISA( ${\left(1+x\right)}^{n}=1+\frac{nx}{1!}+\frac{n\left(n-1\right)x^{2}}{2!}+\cdots$ ). PMA-primed THP-1 macrophages were stimulated with LPS (10 ng/ml) for 4 h and NLRP3 agonists for 2 h. Cell lysates were used for MyD88 detection **(E)**, while cell supernatants were used for active caspase-1 detection. PMA-primed THP-1 macrophages were stimulated with LPS (10 ng/ml) for 4 h and NLRP3 agonists for 2 h and then stimulated again with 10 ng/ml of LPS for 1.5 h (F-H). Supernatants were collected and levels of cytokines determined using ELISA. Data are represented as mean ± SD of at least 3 replicates **(A–D, F–H)**. Experiments were repeated at least three times with similar results. One-tailed unpaired t-test was used for statistical analysis, **p < 0.01, ***p < 0.005.

This far we demonstrated that inflammasome activation dampens LPS signaling, which acts through TLR4 that engages adaptors MyD88 and TRIF to sustain signaling. It had been reported previously that inflammasome effector caspase-1 cleaves TRIF (33). To rule out the effect of the reported TRIF cleavage by caspase-1 and to investigate whether the observed effect is specific for LPS/TLR4 signaling, we monitored the effect of inflammasome activation on TLR2 signaling. PMA-primed THP-1 cells were stimulated with Pam3CSK4, a TLR2 agonist which signals exclusively through MyD88. The observed effect on the cytokine production (**Figures 3A–C**) mirrored the effect of LPS stimulation (**Figure 1**), and the decrease in IL-6 and TNFα has been also reversed by the addition of NLRP3 inhibitor MCC950 (**Figures 3A–C**).

To demonstrate the relevance of the inflammasome-mediated negative regulation on TLR signaling in infection, PMA-primed THP-1 cells were infected with *Staphylococcus aureus*. *S. aureus* activates both TLR2 (34) and the NLRP3 inflammasome (35, 36). In accordance to cell stimulation by purified TLR/NLR agonists, the TNFα production triggered by *S. aureus* infection was increased by caspase or NLRP3 inhibition (**Figure 3D**). Together, these results support the concept that activation of caspase-1 triggered by inflammasome stimulation results in a downregulation of MyD88-mediated signaling in human cells.

We were interested whether the observed effect was due to the cleavage of MyD88 mediated by caspase-1. Western blot analysis of lysates of cells stimulated by LPS in combination with different NLRP3 agonists revealed the presence of C-terminal fragment of MyD88 of less than 20 kDa (**Figure 3E** and **Supplementary Figure 1D**), suggesting a cleavage site within the INT domain of MyD88. Since MyD88-mediated signaling is initiated before the caspase-1 cleavage can occur, the NLRP3 activated caspase-1 could only impair the first signal with some delay and only partly suppress production of MyD88 signaling-mediated cytokines. When cells were stimulated for the second time with LPS after the initial LPS/NLRP3 stimulation, the difference in the produced cytokines increased further, suggesting that the cleaved MyD88 impaired the transmission of the signal by additional TLR stimulation, thereby inducing the tolerance (**Figures 3F–H**).

**Figure 4:**
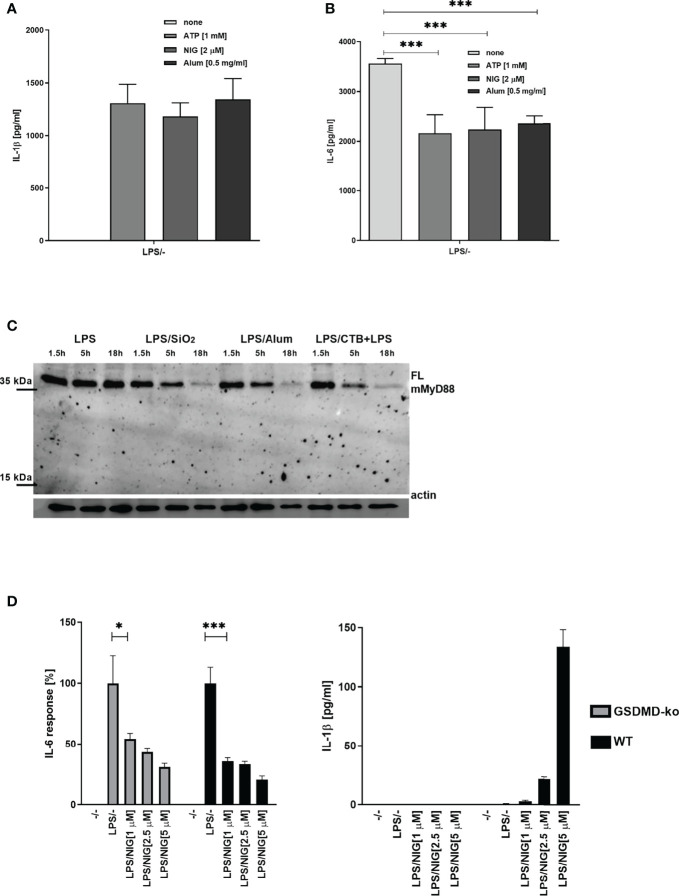
Effect of NLRP3 inflammasome activation on MyD88-dependent cytokines and MyD88 cleavage in mouse cells. Mouse BMDMs were first primed with LPS for 3 h; following this, inflammasome activators (1 mM ATP, 2 μM nigericin, 0.5 mg/ml Alum) were added. After 3 h, supernatants were collected and cytokine concentration was determined using ELISA **(A, B)**. Peritoneal macrophages were primed with LPS for 6 h, after which activators (0.1 mg/ml SiO_2_, 0.5 mg/ml Alum; 20 μg/ml CTB + 2 μg/ml ultrapure LPS) were added. After 1.5 h, 5 h, and 18 h, cells were lysed and blotted for MyD88 **(C)**. Immortalized wt and GSDMD-KO BMDMs were first primed with LPS for 4 h and stimulated with nigericin for 3 h **(D)**. Data are represented as mean ± SD of at least 3 replicates **(A, B, D)**. Representative results of three independent experiments are shown. One-tailed unpaired t-test was used for statistical analysis. *p < 0.05, ***p < 0.005.

We next tested whether cleavage of MyD88 due to inflammasome activation and a decrease in MyD88-dependent cytokines is specific to human cells. A smaller decrease in IL-6 but not IL-1β production after TLR/NLRP3 inflammasome stimulation was also observed in mouse BMDMs (**Figures 4A, B**). Activation of the inflammasome in addition to LPS stimulation led to a decreased amount of full-length MyD88 (**Figure 4C**), but we were unable to detect the cleaved fragment of MyD88. Nigericin-mediated NLRP3 activation can lead to pore formation and pyroptosis that depend on gasdermin D (37–40), through which IL-1β is released (25, 41). While IL-1β release was completely blocked by the GSDMD deficiency (**Figure 4D** right) (25), the decrease in IL-6 response upon nigericin stimulation was comparable in wild-type and GSDMD-deficient macrophages (**Figure 4D** left) which was expected as caspase-1 activity is upstream of GSDMD in inflammasome activation cascade and which further proves that decreased cytokine level is not due to cell pyroptosis.

### Identification of Caspase-1 Cleavage Sites in Human and Murine MyD88

**Figure 5:**
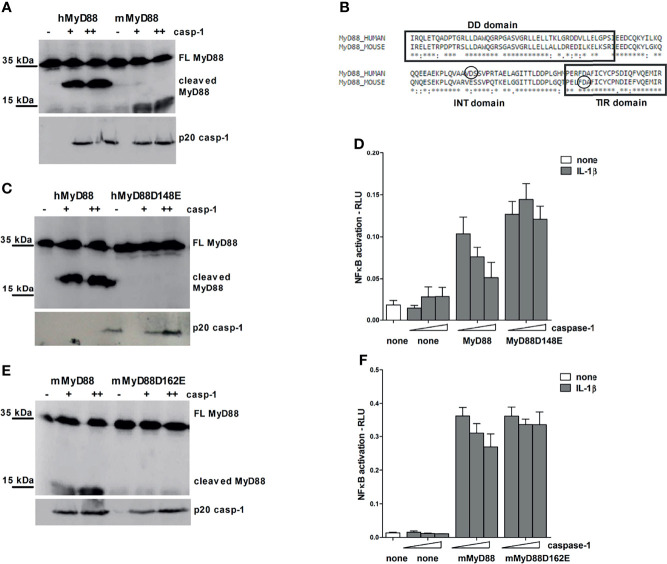
Different caspase-1 cleavage sites in human and mouse MyD88 determine the strength of MyD88-mediated signaling inhibition. HEK293T cells were transfected with human or mouse MyD88 and/or point mutants (0.3 μg or 1 μg) along with increasing amounts of caspase-1 (75/150 ng or 150/300 ng) plasmid. After 24 hours cells were lysed and lysates were blotted for MyD88, while cell supernatants were used for detection of active caspase-1 **(A, C, E)**. Alignment of human and mouse MyD88 with the indicated position of caspase-1 cleavage site **(B)**. MyD88-KO HEK293 cells were transfected with human or mouse MyD88 and/or point mutants (0.05 or 0.1 ng/well) along with increasing amounts of caspase-1 (1/2.5 or 10/25 ng/well) and reporter plasmids. Cells were stimulated with 10 ng/ml of interleukin-1β (IL-1β) for 16 h, lysed and NF-κB activation was measured using luciferase assay. Data are represented as mean ± SD of at least 3 replicates **(D, F)**. Experiments were repeated at least three times with similar results.

To determine the exact cleavage site within MyD88, protein sequences were first analyzed *via* the CASVM server (21). Several caspase-1 cleavage sites were predicted in both human and mouse MyD88; however, the precise positions of the proposed cleavage sites differed between human and mouse MyD88 (**Supplementary Figure 2**). To determine whether the caspase-1 can also cleave mouse MyD88, human or mouse MyD88 was expressed along with the caspase-1 in HEK293T cells (**Figure 5A**). Ectopic overexpression of caspase-1 led to its auto-activation due to oligomerization through CARD domains (42) and resulted in cleavage followed by detection of an activated p20 caspase-1 fragment (**Figure 5A**). Cleavage of human MyD88 by the caspase-1 was observed (**Figure 5**) and it generated a C-terminal fragment of human MyD88 corresponding to the size of the detected MyD88 fragment in THP-1 or PBMCs (**Figure 3E** and **Supplementary Figure 1D**). The C-terminal cleavage fragment was also detected upon coexpression of mouse MyD88 and caspase-1, although the generated fragment was smaller than that of the human MyD88 (**Figure 5A**). The precise cleavage site for caspase-1 is therefore not conserved in human and mouse MyD88 in agreement with the prediction for the caspase-1 cleavage site and sequence alignment (**Supplementary Figure 2**); nevertheless, the target motifs were in both proteins located in close proximity, indicating that the caspase-1 cleavage site functionality was conserved despite the low sequence conservation in this region.

Point mutagenesis of the aspartate residues (D) to glutamate residues (E) was used to pinpoint the precise caspase-1 cleavage site. The cleavage site in human MyD88 corresponds to aspartate 148, whereas in mouse MyD88, the site occurs at aspartate 162 at the beginning of the TIR domain (**Figure 5B**); in agreement to the smaller size of the cleaved C-terminal mouse MyD88 fragment. Point mutants hMyD88^D148E^ and mMyD88^D162E^ in human and mouse MyD88, respectively, made them resistant to the caspase-1 cleavage (**Figures 5C, E**).

In order to gain insight into how different caspase-1 cleavage sites between mouse and human protein affect the MyD88 signaling, wt MyD88 and non-cleavable mutants were introduced into a MyD88 knockout human HEK293 cells. Overexpression of caspase-1 decreased signaling through IL-1R in either human or mouse MyD88 transfected cells but had no effect in cells expressing point mutants hMyD88^D148E^ and mMyD88^D162E^ that were otherwise fully signaling competent (**Figures 5D, F**). The effect of caspase-1 co-transfection on IL-1R signaling was stronger in the case of human compared to mouse MyD88. Since the cleavage site did not completely conform to the consensus motif and for hMyd88 also coincided with caspase-3 cleavage site (43), a caspase-1 cleavage sequence from pro-IL-1β was introduced into the same position in hMyD88. Nevertheless, a replacement of the original sequence with the optimal caspase-1 cleavage site did not change the cleavage efficiency (**Supplementary Figure 3**), suggesting that the caspase-1 cleavage site in human MyD88 is sufficient for caspase-1 processing and is therefore likely to be of physiological importance.

### Inhibitory Potential of the Caspase-1 Cleavage Products of MyD88

Cleavage of MyD88 within the INT domain resulted in two protein fragments comprising separated DD and TIR domain which are therefore unable to recruit IRAK4 to the dimerized TLRs. On the other hand, the released TIR domain of MyD88 might be able to inhibit signaling *via* binding to the TIR domain of activated TLRs, thereby preventing recruitment of full length MyD88 as shown for TIR domains before (11, 44). Interestingly, we found that the C-terminal TIR domain of human *versus* mouse MyD88 resulting from caspase-1 cleavage had different effects on the TLR4/MD-2 signaling due to differences in the position of the cleavage site. The more extensively truncated mouse C-terminal MyD88 domain inhibited signaling to a significantly lower degree than longer human cleavage product (**Supplementary Figure 4A**). In addition, the TIR fragment of mouse MyD88 seems to be unstable (**Supplementary Figure 4B**), similarly to detection of the endogenous MyD88 in mouse cells, where we were unable to detect the cleaved fragment, suggesting its degradation, presumably due to the destabilized TIR domain.

To further explore the differences in the caspase-1 cleavage sites between human and mouse MyD88, a mutant containing a human cleavage site (148AAVD) combined with the mutated initial cleavage site (D162E) in the mouse MyD88 was generated (mMyD88^(148AAVD)D162E^). The mMyD88^(148AAVD)D162E^ mutant gained a new functional caspase-1 cleavage site, which was confirmed by the detection of a fragment corresponding to the size of a human cleaved MyD88 fragment in cells, coexpressing mutant mMyD88^(148AAVD)D162E^ and caspase-1 (**Supplementary Figure 4C**). The introduction of a human cleavage site into mouse MyD88 also increased the inhibitory effect of caspase-1 on IL-1R signaling in MyD88KO HEK293 cells (**Supplementary Figure 4D**), corroborating the functional difference caused by different position of the cleavage site within MyD88.

### *In Vivo* Inflammasome-Mediated Negative Regulation of MyD88 Signaling

**Figure 6:**
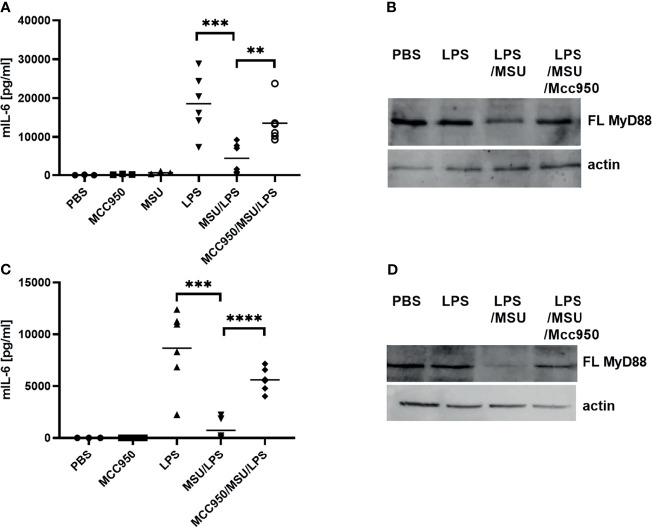
LPS/MSU challenge of animals results in decreased cytokine levels in comparison to LPS stimulation. Activation of the inflammasome was triggered in wild-type (wt) **(A, B)** or *Trif*-/- knockout (KO) **(C, D)** mice by the intraperitoneal injection of MSU at 1 mg/animal. Optionally, the NLRP3 inhibitor MCC950 was given intraperitoneally 1 h prior to MSU. Two hours after MSU injection, animals were administered 0.75 mg/kg LPS intraperitoneally. Four hours later, blood was collected for cytokine detection using ELISA (A, C), while peritoneal lavage obtained cells were used for MyD88 detection (B, D). Unpaired two-tailed t-test with Welch’s correction was used for comparison of TRIF-KO populations treated with LPS and MSU/LPS and unpaired two-tailed t-test was used otherwise. **p < 0.01, ***p < 0.005.

To determine whether the observed negative regulation of MyD88 by the inflammasome activation could have a physiological relevance, NLRP3 inflammasome was activated in mice using monosodium urate crystals (MSU). MSU alone did not induce production of IL-6 (**Figure 6A**), but significantly decreased the IL-6 production in combination with LPS (**Figure 6A**) as expected based on cell culture results. Also in accordance with results on cell cultures, co-administration of the NLRP3 inhibitor MCC950 reversed the effect of MSU on IL-6 production. The analysis of MyD88 in peritoneal macrophages (**Figure 6B**) clearly showed a decreased amount of the full-length MyD88 in LPS/MSU animals in comparison to LPS injected animals. Similar results were obtained in TRIF knockout (KO) animals, confirming the important role of MyD88 in this animal model as the target of caspase-1 mediated cleavage (**Figures 6C, D**).

### Proteolytic Cleavage of MyD88 in Waldenström’s Macroglobulinemia

**Figure 7:**
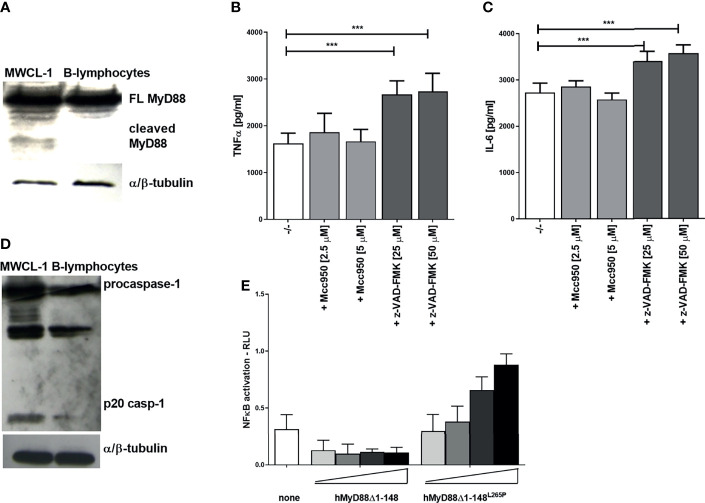
Constitutively present MyD88 cleavage fragment in the Waldenström’s macroglobulinemia (WM) cell line and lack of signaling inhibition by TIR^L265P^. MWCL-1 cells or B-lymphocytes were lysed and blotted for MyD88 **(A)** or caspase-1 **(D)** detection. MWCL-1 cells were seeded into fresh media, and inhibitors were added to the cells for 24 h. Supernatants were collected and the amounts of cytokines determined using ELISA. Data are represented as mean ± SD of at least 3 replicates **(B, C)**. HEK293 cells were transfected with MyD88 cleavage fragments (hMyD88Δ1-148 or hMyD88Δ1-148^L265P^; 1, 5, 10, 25 ng/well) and reporter plasmids. Cells were lysed after 16 h and NF-κB activation was measured using luciferase assay. Data are represented as mean ± SD of at least 3 replicates **(E)**. Experiments were repeated at least three times with similar results. One-tailed unpaired t-test was used for statistical analysis, ***p < 0.005.

Dysregulated and constitutively active MyD88 signaling caused by the mutation L265P within its TIR domain is characteristic for distinct types of diffuse large B-cell lymphoma (e.g., Waldenström’s macroglobulinaemia, WM), where almost 90% of patients harbor L265P mutation (8, 9). MyD88 signaling represents a survival signal for this type of cancer. In principle, inhibition of MyD88-mediated signaling by caspase-1 cleavage might be able to inhibit cancer growth. Therefore the presence of cleaved MyD88 was tested in the WM cell line MWCL-1, established from the bone marrow aspirate of a WM patient which harbors the constitutively active MyD88^L265P^ mutation (26). A cleaved fragment of MyD88 was indeed observed in nonstimulated MWCL-1 lymphoblastoma cells in contrast to normal B-lymphocytes (**Figure 7A**). The WM cell line constitutively produces a substantial amount of proinflammatory cytokines; however, caspase inhibition only further increased the production of TNFα and IL-6 in MWCL-1 (**Figures 7B, C**). Interestingly, the NLRP3 inhibitor had no significant effect on cytokine production in WM cell line (**Figures 7B, C**), suggesting NLRP3-independent caspase-1 maturation. It has been reported that the WM cell line exhibits elevated transcription of the procaspase-1 (45). We showed that caspase-1 not only was expressed at higher level but was also processed into an active form (**Figure 7D**), thereby explaining MyD88 cleavage in WM cells independently of NLRP3. The contribution of the TIR domain released by cleavage of the full-length MyD88 to the signaling pathway may however have different effect in case of L265P mutation than in cells harboring the wt MyD88. We determined previously that a TIR^L265P^ domain is also able to recruit mutated or wt full length MyD88 due to its oligomerization propensity and support constitutive activation of the MyD88 signaling pathway (46). This implies that the caspase-1 cleaved product of MyD88^L265P^ might not inhibit MyD88-mediated signaling but rather sustain it. To check this hypothesis, the effect of the C-terminal fragment of MyD88 corresponding to the cleaved protein was investigated. We found that the hMyD88Δ1-148^L265P^ fragment indeed exhibited constitutive activity in the presence of full length MyD88 (**Figure 7E**); thus revealing that the lymphoma cells harboring the L265P mutation are able to evade the caspase-mediated negative feedback regulation of the MyD88-mediated inflammatory signaling pathway.

## Discussion

**Figure 8:**
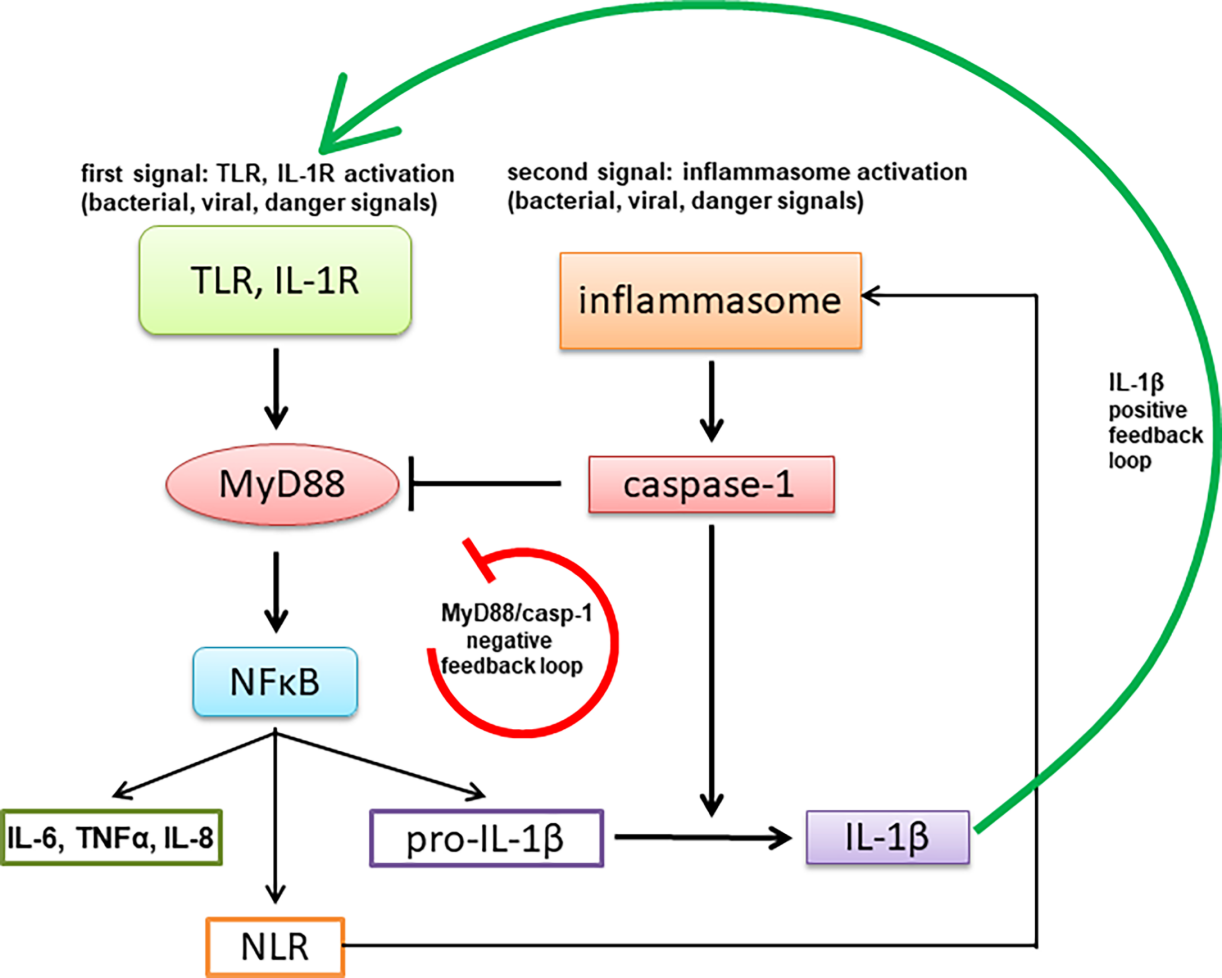
A schematic representation of negative feedback regulation of MyD88-mediated signaling through activation of the inflammasome.

Tight regulation of signaling through MyD88 is highly important for proper functioning of innate immunity as it mediates signaling through TLRs and IL-1R, thus affecting also MyD88 signaling through inflammasomes that results in IL-1β production. Impairment of MyD88 signaling therefore also indirectly inhibits inflammasome-mediated response. The described caspase-1-mediated MyD88 cleavage and release of the inhibitory TIR domain represents a negative feedback loop, in contrast to the other reported effects of caspase-1 cleavage of signaling mediators, such as Mal/TIRAP, TRIF and cGAS, that inhibit a parallel signaling pathway (33, 47–50) (**Figure 8**). The caspase-1 cleavage site is specific as a single point mutation of the target Asp to the biochemically conserved Glu residue completely abolished cleavage in contrast to other targets, such as cGAS (50), where several sites had to be mutated.

Comparison of mouse and human inflammasome signaling and evolutionary aspects of it are a part of lively research (51–54). The effect of cleavage effect exhibits some differences between human and mouse MyD88, which may be an evolutionary adaptation of mouse and some other rodent species to tune the appropriate immune response. This difference is likely of functional importance since, although the particular sequence and position of the cleavage site are not conserved, they are conserved at the functional level. Further, the replacement of mouse MyD88 with human cleavage site and position modified the functional effect of caspase-1 cleavage. It is interesting to note that the cleavage between the DD and TIR domains of MyD88 exerts a dual inhibitory function—to inactivate the full-length MyD88 and to generate an inhibitory TIR domain (11, 55), which potentiates the inhibition of the MyD88 signaling pathway. The downregulation of cytokine response to TLR agonists was strengthened in case of repeated TLR stimulation, which contributes to the tolerance (56). We demonstrated inhibitory effects on MyD88 signaling downstream of TLR2/TLR1, TLR4 and IL-1R, indicating that signaling of other TLR that act through MyD88 such as TLR7 and TLR9 is likely to be similarly affected, but not TLR3 that acts through adaptor TRIF. Based on the observed differences in the amount of the cleaved MyD88 (**Figure 3**, **5**) we speculate that it is likely that only MyD88 that has not been already engaged in the tightly packed myddosomal complex may be sensitive to the caspase-1 cleavage. The position of the cleavage site within MyD88 orthologues governs the inhibitory potency of the released TIR domains being more potent for human than mouse MyD88 cleavage product, where the proteolytic cleavage site, shifted towards the C-terminus, leads to the destabilized TIR domain and may make it prone to degradation. While B-cell lymphoma cells with constitutively active MyD88^L265P^ contain the cleaved MyD88 fragment in the absence of additional stimulation, those cells have a unique ability to escape this negative regulation mechanism, as the mutated TIR^L265P^ domain resulting from the proteolytic cleavage is no longer inhibitory and can in contrast even support signaling in the presence of full-length MyD88. Since some caspase and NLRP3 inhibitors are already in clinical trials (57), our findings indicate that they may trigger effects on innate immune response due to differences in regulation of cellular signaling pathways among different types of cells, diseases and animal species.

The two-stage activation of the inflammasome is an example of a tight regulation of the production of IL-1β that is processed by caspase-1. The IL-1β signaling pathway forms a positive feedback loop, mediated by MyD88 (58). Positive feedback loops are inherently prone to strong amplification with potential deleterious effects. Therefore, the function of the negative feedback loop impeding activation of IL-1R and TLR signaling *via* MyD88 regulated by caspase-1 activity likely serves as an additional mechanism to prevent the excessive inflammation. Our results may explain the findings from studies of autoinflammatory disorders, such as familial cold autoinflammatory syndrome (FCAS), which is characterized by mutations in the NLRP3 gene that result in augmented caspase-1 activation and elevated production of IL-1β, leading to episodic arthralgia, rash, and fever in response to cold exposure. Secretion of IL-6 and IL-1α in response to LPS, however, is reduced in PBMCs from FCAS patients (59). Similar attenuation was observed for IL-1α and TNFα after LPS stimulation in haplotype 2A patients, which correlates with mutations in the NLRP1 gene that are associated with a high risk of autoimmune diseases, such as vitiligo, Addison’s disease, and type 1 diabetes (60). The observed attenuation of MyD88-dependent cytokines could be a consequence of the negative feedback loop, as demonstrated in this report.

In conclusion, inflammasome-mediated negative feedback regulation of MyD88 signaling likely has an important role for tuning of the immune response, where the time delay between the initiation of the first signal and the onset of inhibition may be also important in retaining the responsiveness of the system in conjunction with the ability to limit the excessive response.

## Data Availability Statement

The raw data supporting the conclusions of this article will be made available by the authors, without undue reservation.

## Ethics Statement

The studies involving human participants were reviewed and approved by Republic of Slovenia National Medical Ethics Committee. The patients/participants provided their written informed consent to participate in this study. The animal study was reviewed and approved by Administration of the Republic of Slovenia for Food Safety, Veterinary Sector and Plant Protection of the Ministry of Agriculture, Forestry and Foods, Republic of Slovenia.

## Author Contributions

MA performed and analyzed the experiments on THP-1, HEK, and MWCL-1 cells; discussed the results; and wrote the manuscript. IH-B performed experiments on mouse macrophages, wrote the manuscript and discussed the results. DL performed animal experiments. TP, BG, and UP performed experiments on PBMCs. GP made MyD88 knockout HEK293 cells. MM-K performed ELISA assays. ST provided reagents and discussed the results. RJ conceived the idea, discussed the results, and wrote the manuscript. All authors contributed to the article and approved the submitted version.

## Funding

This work was funded by the projects and program from the Slovenian Research Agency (Z3-6786, J3-6804, J3-1746, J3-9258 and P4-0176) and from ICGEB CRP SVN18-01.

## Conflict of Interest

The authors declare that the research was conducted in the absence of any commercial or financial relationships that could be construed as a potential conflict of interest.

## Publisher’s Note

All claims expressed in this article are solely those of the authors and do not necessarily represent those of their affiliated organizations, or those of the publisher, the editors and the reviewers. Any product that may be evaluated in this article, or claim that may be made by its manufacturer, is not guaranteed or endorsed by the publisher.
