# Supplementary material for: Cleavage-Mediated Regulation of Myd88 Signaling by Inflammasome-Activated Caspase-1
Source: Front Immunol. 2022 Jan 5;12:790258. doi: 10.3389/fimmu.2021.790258 (PMC8767097; doi:10.3389/fimmu.2021.790258)
Supplement: Supplementary file 1 [file DataSheet_1.docx]

Supplementary Material


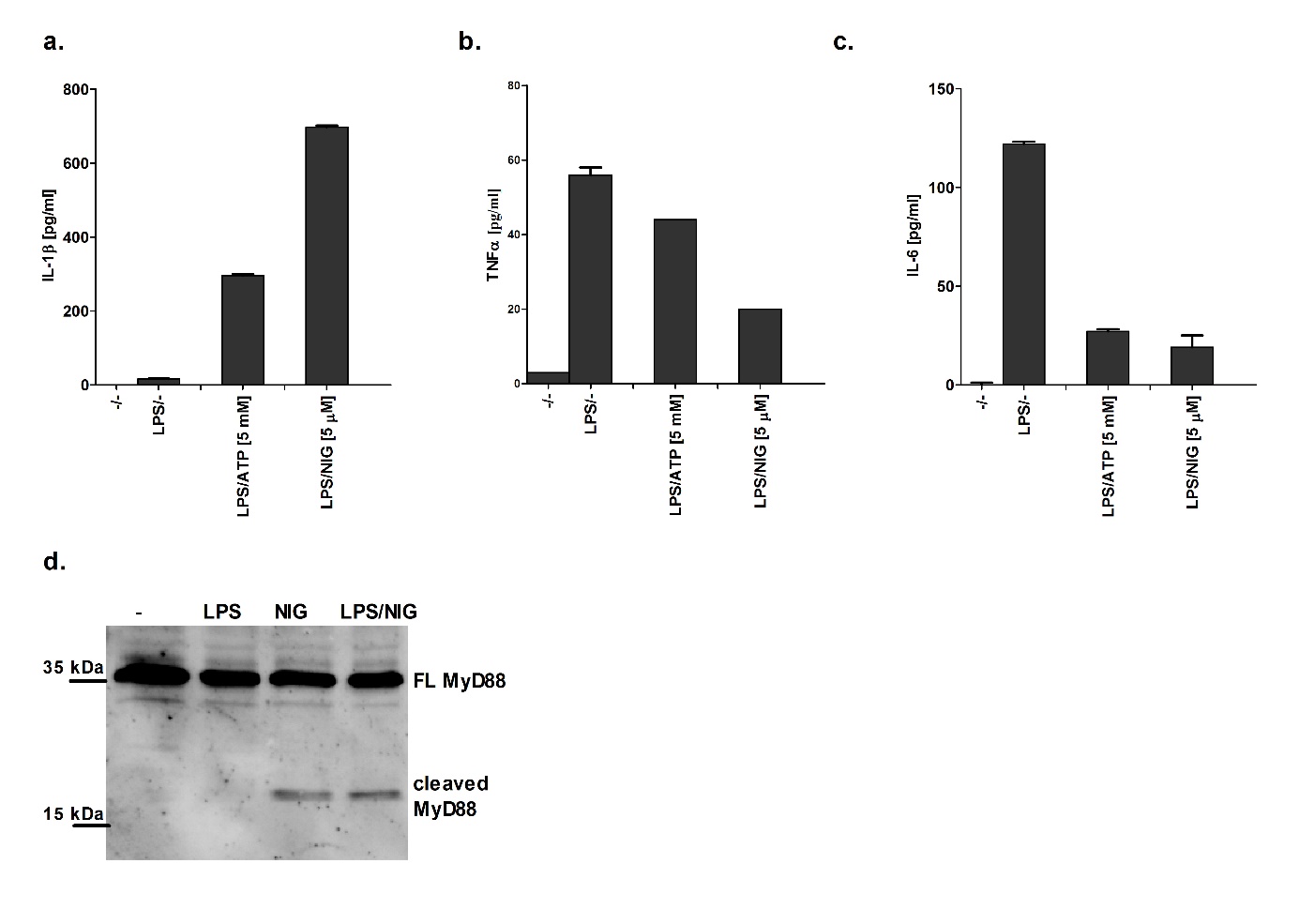


**Supplementary Figure 1.** Activation of NLRP3 inflammasome decreases the production of TNFα and IL-6, coinciding with the cleavage of MyD88 in human PBMCs.

PBMCs were stimulated with LPS (10 ng/ml) for 2 h and then with NLRP3 agonists for 6 h. Supernatants were collected and the levels of cytokines determined using ELISA. Data are represented as mean ± SD of at least 3 replicates (a–c). Cells were lysed and used for MyD88 detection (d).


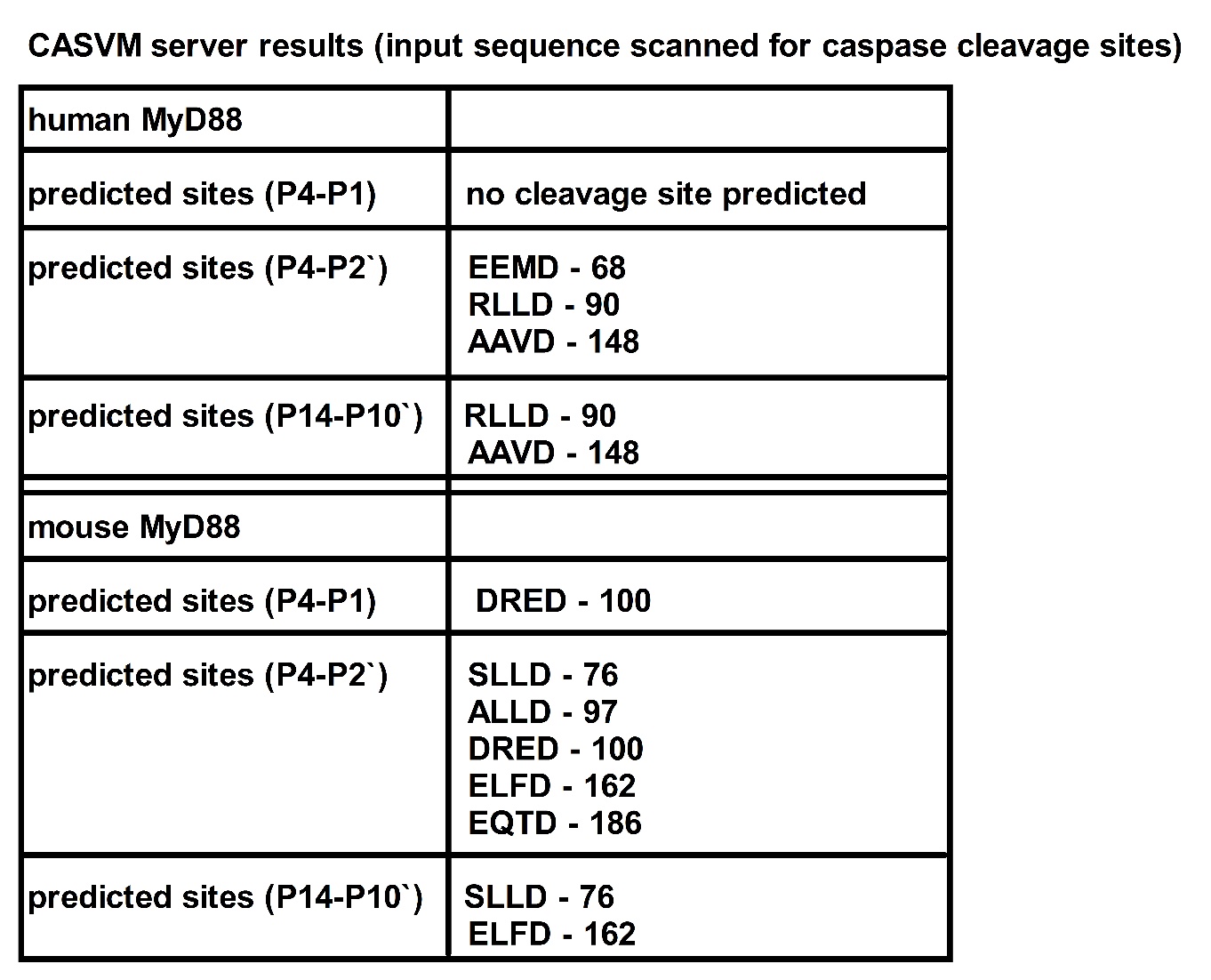


**Supplementary Figure 2.** CASVM server prediction of potential caspase-1 cleavage sites in human and mouse MyD88.


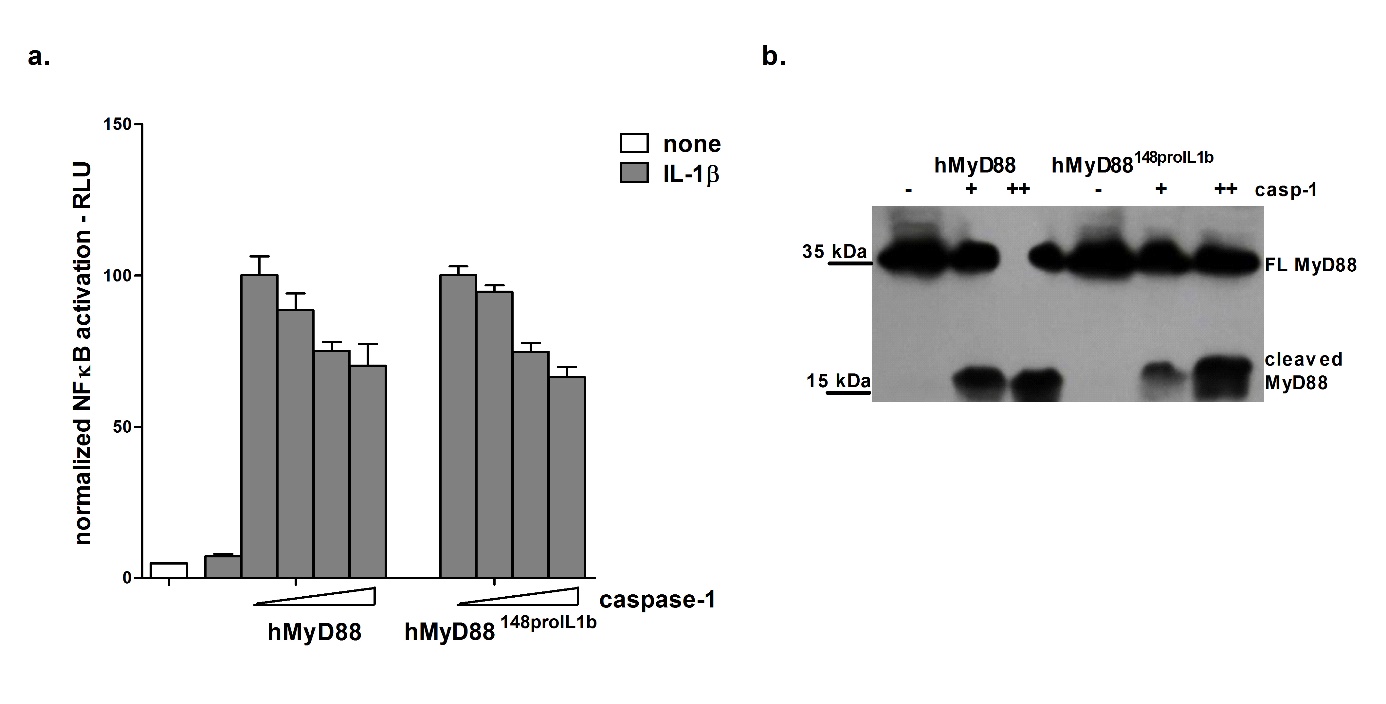


**Supplementary Figure 3.** Insertion of ideal caspase-1 recognition sequence in hMyD88 is comparable to wild-type (wt) MyD88 regarding the effect of caspase-1 co-transfection on interleukin-1R (IL-1R) signaling and MyD88 cleavage.

MyD88KOHEK293 cells were transfected with wt hMyD88 or hMyD88^148proIL1b^ along with increasing amounts of caspase-1 (1 or 2.5 ng/well) and reporter plasmids. Cells were stimulated with 10 ng/ml of IL-1β for 16h, lysed and NF-κB activity was measured using luciferase assay. Data are represented as mean ± SD of at least 3 replicates (a). HEK293T cells were transfected with wt hMyD88 or hMyD88^148proIL1b^ along with increasing amounts of caspase-1. Cell lysates were blotted for MyD88 (b). Experiments were repeated at least three times with similar results.


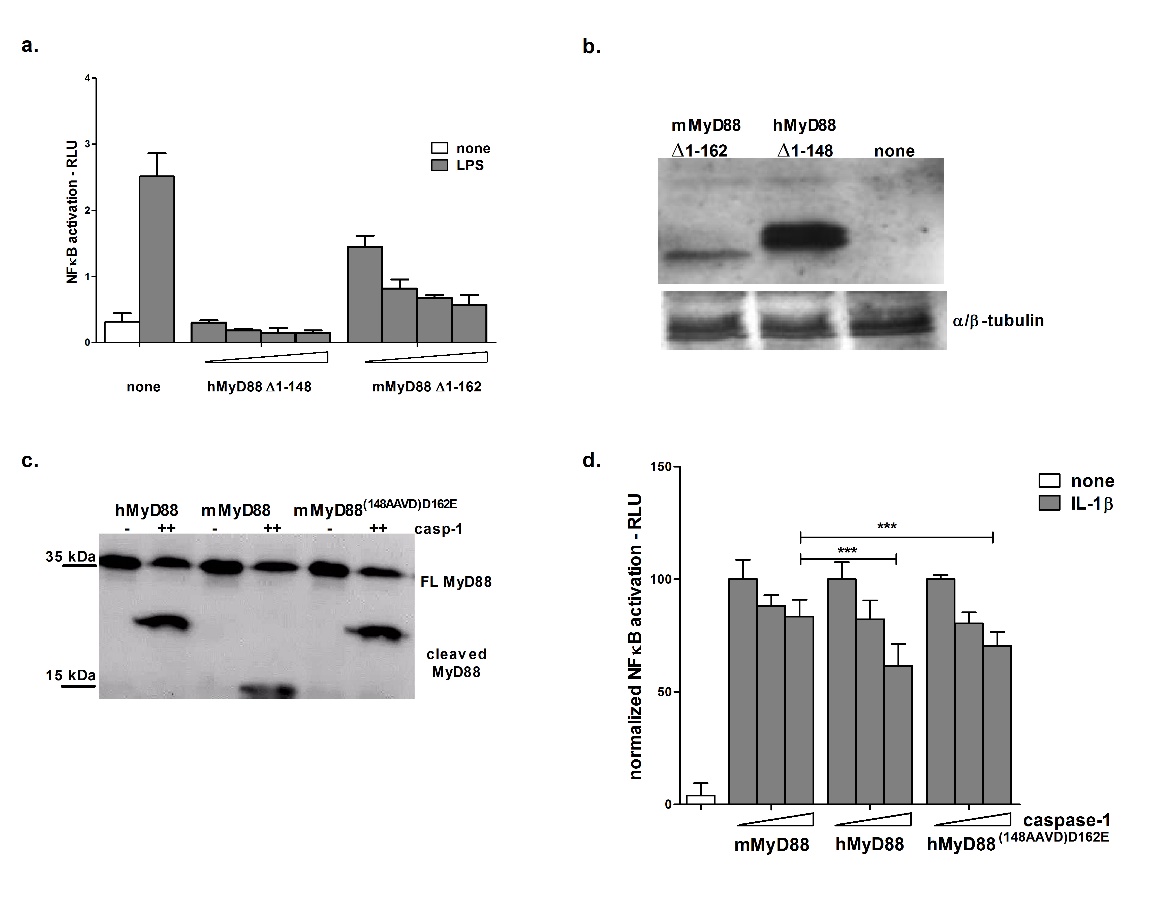


**Supplementary Figure 4:** Caspase-1 cleavage sites in human and mouse MyD88 result in cleavage fragments with different size and inhibitory potential.

HEK293 cells were transfected with toll-like receptor-4 (TLR4)/MD-2 and MyD88 cleavage fragments (hMyD88Δ1-148 or mMyD88Δ1-162; 1, 5, 10 or 25 ng/well) with reporter plasmids. Cells were stimulated with 20 ng/ml of LPS for 16h, lysed and NF-κB activity was measured using luciferase assay. Data are represented as mean ± SD (a). HEK293T cells were transfected with MyD88 cleavage fragments (hMyD88Δ1-148 or mMyD88Δ1-162) (b) or human or mouse MyD88 or mutant along with increasing amounts of caspase-1 (c). Cell lysates were blotted for MyD88 (b, c). MyD88KOHEK293 cells were transfected with human or mouse MyD88 or mutant along with increasing amounts of caspase-1 (1 or 2.5 ng/well) and reporter plasmids. Cells were stimulated 6 h later with 10 ng/ml of interleukin-1β (IL-1β), lysed after 16 h and luciferase assay measured. Data are represented as mean ± SD of at least 3 replicates (d). Experiments were repeated at least three times with similar results. One-tailed unpaired t-test was used for statistical analysis, *** *p* < 0.005.
